# The Surgical Management of Cerebral Arteriovenous Malformations: A Retrospective Analysis of Outcomes and Complications

**DOI:** 10.7759/cureus.89498

**Published:** 2025-08-06

**Authors:** Saddam Hussain, Abdul Moez, Aiza Ali Akbar, Syeda W Batool, Amna Akbar, Amir Iqbal Ali, Muhammad Rizwan Umer, Amna Sahar

**Affiliations:** 1 Neurosurgery, Combined Military Hospital, Rawalakot, PAK; 2 Neurosurgery, Ghurki Hospital, Lahore, PAK; 3 Department of Cardiology, DHQ Teaching Hospital, Mirpur, PAK; 4 Department of General Surgery, DHQ Teaching Hospital, Mirpur, PAK; 5 Department of General Surgery, Pilgrim Hospital, Boston, GBR; 6 General Surgery, Combined Military Hospital, Muzaffarabad, PAK; 7 Trauma Surgery, Royal Sussex County Hospital, Brighton, GBR; 8 Medicine, Fatima Jinnah Medical University, Lahore, PAK

**Keywords:** cerebral arteriovenous malformations, endovascular embolization, hypertension, kaplan-meier survival analysis, microsurgery, post-surgical complications, predictors of complications, radiosurgery, recovery, surgical management

## Abstract

This study aimed to investigate the surgical management of cerebral arteriovenous malformations (AVMs) by analyzing clinical outcomes and complications in 600 patients (100%) who underwent surgery. The mean age of the cohort was 36.7 years (SD = 12.3), with a majority being female (n = 315, 52.5%). Patients were classified by AVM size: small (35.5%, n = 213), medium (31.5%, n = 189), and large (33%, n = 198). Surgical approaches included microsurgery (31.3%, n = 188), endovascular embolization (33.5%, n = 201), and radiosurgery (35.2%, n = 211). Surgical outcomes revealed that 33.8% (n = 203) achieved complete resection, 32.7% (n = 196) had partial resection, and 33.5% (n = 201) underwent no resection. Post-surgical complications were experienced by 51.5% (n = 309) of patients, with the most common being postoperative hemorrhage (15.2%, n = 91), infection (14.8%, n = 89), and seizures (9.8%, n = 59). Logistic regression identified AVM size (OR = 2.3, *p* = 0.01), hypertension (OR = 1.8, *p* = 0.03), and seizure history (OR = 1.7, *p* = 0.04) as significant predictors of complications. Kaplan-Meier analysis revealed that patients with complete resection had the highest one-year recovery rate (78%), compared to partial (48%) and no resection (38%). Hospital stay was significantly longer for large AVMs (mean = 18.5 days) versus small AVMs (mean = 12.3 days; *p* = 0.001). These findings underscore the importance of AVM size and patient comorbidities in predicting surgical outcomes and complications, highlighting the need for tailored treatment approaches.

## Introduction

Cerebral arteriovenous malformations (AVMs) are congenital vascular anomalies characterized by direct connections between arteries and veins without an intervening capillary bed, resulting in abnormal hemodynamics and a predisposition to rupture [1,2]. Although rare, AVMs are estimated to affect 0.1-0.5% of the general population, with an annual hemorrhage risk of approximately 2-4% per patient year [3,4]. Many AVMs remain asymptomatic until rupture, often presenting with sudden intracranial hemorrhage, seizures, or focal neurological deficits [2]. Hemorrhage is the initial presentation in about half of all diagnosed cases, and AVMs account for nearly 1% of strokes, predominantly affecting individuals aged 20-40 years [4].

Despite significant advances in diagnosis and intervention, AVMs continue to pose significant management challenges due to the variability in size, location, and vascular architecture. A cornerstone of AVM assessment is the Spetzler-Martin (SM) grading system, which stratifies surgical risk based on lesion size, venous drainage pattern, and eloquence of brain location [5,6]. Although widely used to predict surgical morbidity, this grading system remains underutilized in many retrospective outcome studies, especially in developing regions [5,7].

Surgical treatment of AVMs has evolved significantly over the decades, progressing from high-risk craniotomies to multimodal strategies that include microsurgical resection, endovascular embolization, and radiosurgery [5,8]. Improved imaging techniques such as cerebral angiography, CT, and MRI have enhanced preoperative planning and intraoperative precision, contributing to reduced operative risks [6,9]. Nonetheless, postoperative complications such as hemorrhage, infection, prolonged neurological deficits, and seizures continue to be substantial concerns, especially in high-grade or eloquently located AVMs [10,11]. While several outcome studies have been published from high-income countries, there is a distinct lack of region-specific data from South Asia evaluating surgical success, complication rates, and long-term recovery in large AVM cohorts [12,13]. Moreover, very few studies comprehensively incorporate patient-specific risk factors, such as hypertension or seizure history, into outcome prediction models for surgical AVM management [14].

Given these gaps in the literature, the present study aims to retrospectively evaluate the surgical management of cerebral AVMs in 600 patients treated at a tertiary care center. The objectives include assessing the efficacy of different surgical modalities, determining the incidence and predictors of complications, examining the influence of AVM characteristics and comorbidities on surgical outcomes, and evaluating recovery over short- and long-term follow-up. By incorporating key variables such as AVM size, comorbidity burden (e.g., hypertension, seizure history), and outcome metrics using robust statistical methods, including logistic regression and survival analysis, this study seeks to provide evidence that can inform more personalized and risk-adjusted treatment approaches, particularly in resource-limited healthcare systems.

## Materials and methods

Study design

**Figure 1:**
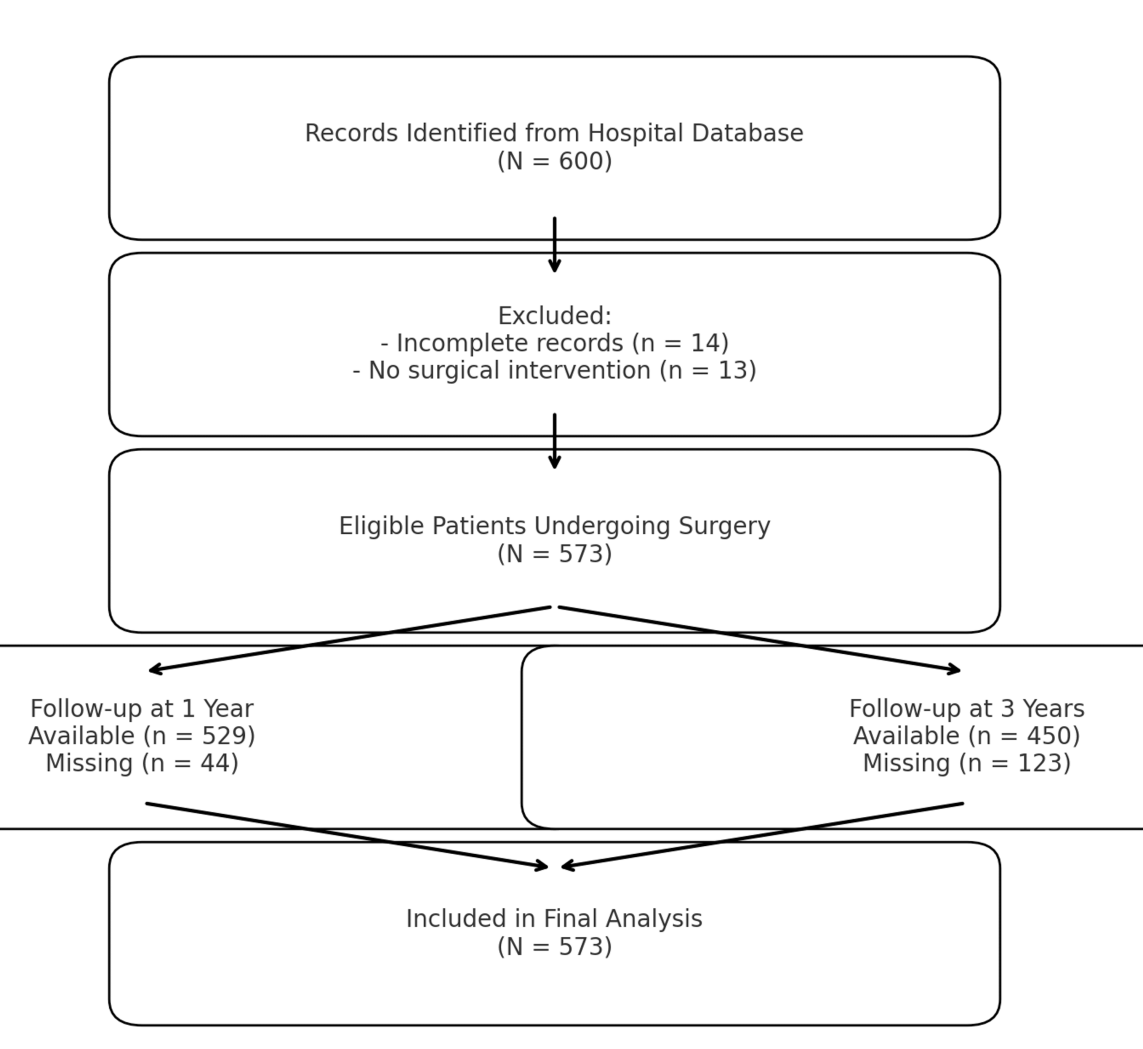
STROBE-style patient flow diagram depicting patient selection STROBE: Strengthening the Reporting of Observational studies in Epidemiology

This was a multicenter retrospective observational study to determine the surgical outcomes and complications of the surgical management of cerebral AVM. Data were obtained from a hospital-wide database containing 600 patients. The focus of the analysis was on surgical outcome, complications, and recovery between one and three years after surgical management. The study adhered to STROBE (Strengthening the Reporting of Observational studies in Epidemiology) reporting guidelines (Figure 1).

Inclusion and exclusion criteria

The inclusion criteria were as follows: patients with a diagnosis of cerebral AVMs who were surgically treated; aged 18-80 years; a diagnosis of cerebral AVMs based on imaging (MRI and CT); and treatment with surgery, which may have included microsurgery, endovascular embolization, or radiosurgery. Patients who received only conservative or medical management without surgical intervention were excluded, as were those with incomplete operative records or follow-up data under one year. Of the 600 patients identified, 27 were excluded from long-term recovery analysis due to missing follow-up documentation.

Demographic and clinical characteristics

Demographic and clinical data for all patients, such as age, sex (female/male), and ethnicity (all were of Pakistani origin), were recorded. Smoking status, alcohol consumption, and comorbidities such as hypertension, diabetes, seizure history, and neurological deficits were also noted. Clinical data on the AVM itself, such as size, location (e.g., brainstem, cerebellum, cerebral cortex, deep brain), and hemorrhage status, were also collected. AVM size classification was independently determined by two board-certified neurosurgeons using MRI/CT imaging and categorized as small (<3 cm), medium (3-6 cm), or large (>6 cm). Any discrepancies were resolved through consensus review.

Surgical treatment and outcomes

The surgical interventions that were performed are microsurgery (the direct resection of an AVM), endovascular embolization (the reduction in the size of an AVM using embolic agents), and radiosurgery (noninvasive radiation therapy for small or deep AVMs). The results of the surgical procedures were classified into three categories: complete resection (no residual AVM on postoperative angiography or MRI), partial resection (residual nidus or shunt flow remaining after intervention), and no resection (surgery was initiated but no AVM tissue was removed due to inaccessibility, intraoperative complications, or preoperative intent to perform palliative embolization only). Surgical outcomes and resection categories were confirmed by operative reports and radiologic findings, and were reviewed independently by both the operating surgeon and a second evaluator. Postoperative complications were recorded, including hemorrhage, infection, and seizures.

Follow-up data

Post-surgical follow-up data were collected for up to three years. Key follow-up variables included hospital stay duration, ICU stay duration, the need for rehabilitation, and long-term recovery. Long-term recovery was classified into three categories: complete recovery, partial recovery, and no recovery. Follow-up data were available for 92.3% of patients at one year and 78.6% at three years. Missing data were handled using complete case analysis without imputation. Sensitivity analysis revealed no significant demographic or clinical differences between those with and without full follow-up.

Statistical analysis

Data were analyzed using SPSS Statistics version 27.0 (IBM Corp., Armonk, NY). Descriptive statistics, including means, medians, and frequencies, were used to summarize demographic and clinical characteristics. Statistical comparisons between groups were made using chi-square tests for categorical variables (e.g., surgical outcomes and AVM size), independent samples t-tests for continuous variables (e.g., hospital stay duration), and one-way ANOVA for comparing ICU stay duration across AVM sizes. Logistic regression analysis was performed to evaluate the relationship between predictors (e.g., age, AVM size, comorbidities) and postoperative complications. Kaplan-Meier survival analysis was conducted to examine time-to-event data, specifically for time to recovery or recurrence of AVMs.

Ethical considerations

This study was conducted as a multicenter retrospective observational study, with the primary site for data collection being Combined Military Hospital (CMH)/HH Sheikh Khalifa bin Zayed Al Nahyan Hospital, Muzaffarabad. Supplementary data were also obtained from the Abbas Institute of Medical Sciences (AIMS). The study was approved by the Institutional Review Board (IRB) at Abbas Institute of Medical Sciences (no: 1610/AIMS/2024). Informed consent was waived due to the retrospective nature of the study, which was conducted between January 5, 2023, and April 22, 2024. All patient data were anonymized before analysis to ensure confidentiality and compliance with ethical research standards.

## Results

Descriptive statistics

**Figure 2:**
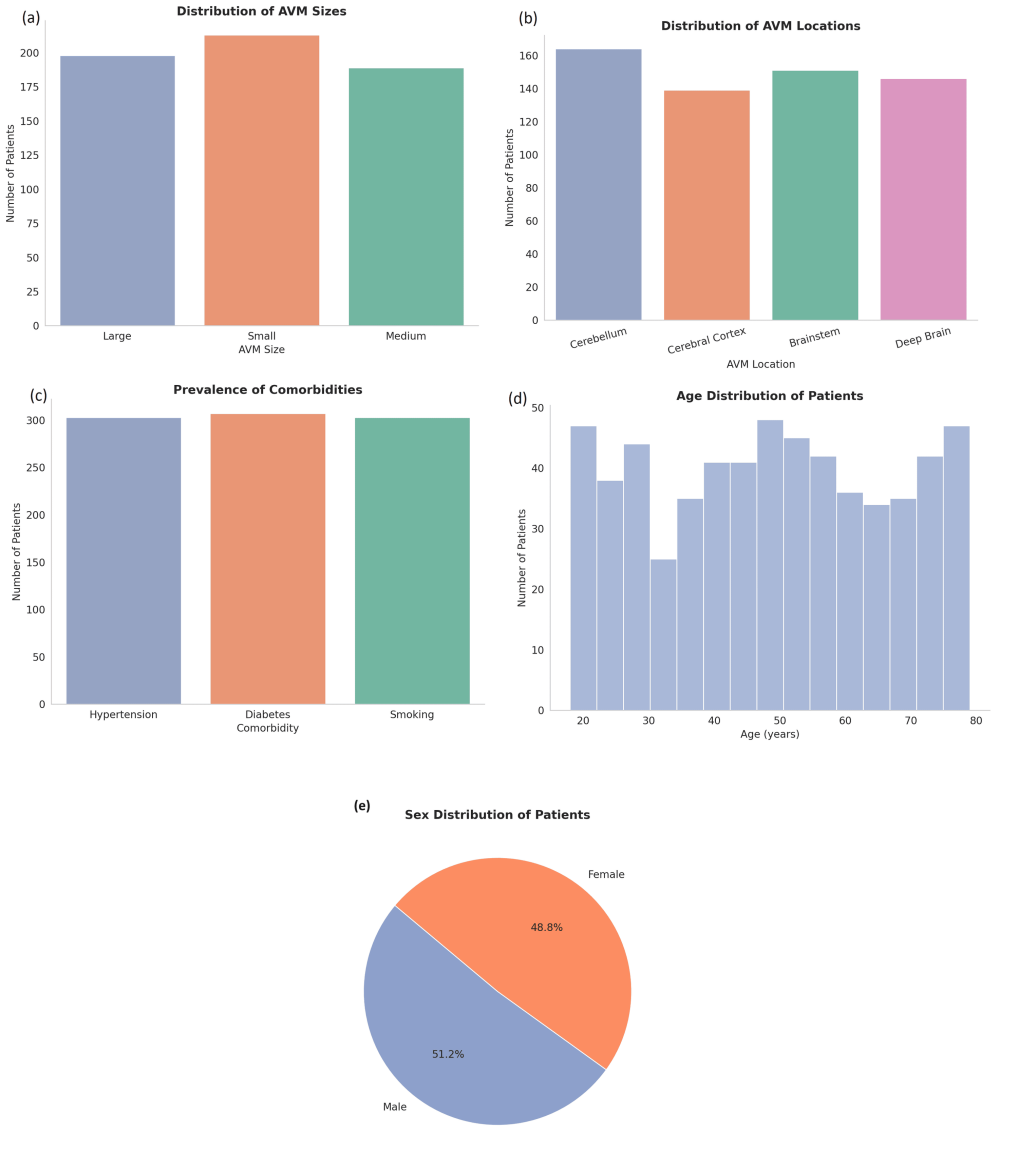
Baseline demographic and clinical characteristics of patients with cerebral AVMs (a) Distribution of AVM sizes reveals a slightly higher number of patients with small AVMs, followed by large and medium-sized lesions. (b) Location-wise, AVMs are most frequently observed in the cerebellum, with relatively balanced representation in the brainstem, deep brain, and cerebral cortex. (c) Prevalence of comorbidities shows nearly equal distribution across hypertension, diabetes, and smoking, each affecting a significant portion of the cohort. (d) Age distribution indicates patients are spread across a broad age range (18–80 years), with modest peaks in the 20s and 60s. (e) Sex distribution shows a near-even split between male (51.2%) and female (48.8%) patients AVM: arteriovenous malformation

This study included a total of 600 patients (100%) who underwent surgical treatment for AVMs. The mean age of the patients was 36.7 years (SD = 12.3; range: 18-79 years. Of the total cohort, 47.5% were male (n = 285) and 52.5% were female (n = 315). Family history of AVMs was reported in 48.3% (n = 290) of the patients, while the remaining 51.7% (n = 310) had no family history. The prevalence of comorbidities was notable, with hypertension affecting 40.5% (n = 243) of patients, diabetes observed in 18.6% (n = 112), and smoking reported by 33.5% (n = 201) of the cohort. The size of the AVMs was categorized as small in 35.5% (n = 213) of patients, medium in 31.5% (n = 189) of patients, and large in 33% (n = 198) of patients. The AVMs were located in the cerebellum (27.3%, n = 163), brainstem (25.2%, n = 151), deep brain regions (24.3%, n = 146), and cerebral cortex (23.2%, n = 138). Additionally, hemorrhage history was reported in 50.2% (n = 301) of patients, and seizures were present in 51% (n = 306) of patients (Figure 2).

Table 1 presents the Chi-square test results for the association between variables. 

**Table 1:** Chi-square test results for the association between variables Chi-square test was used to assess associations between categorical variables. P-values<0.05 were considered statistically significant and are marked with an asterisk (*). Highly significant results with p<0.001 are noted explicitly AVM: arteriovenous malformation; ICU: intensive care unit

Comparison	Degrees of freedom (df)	Chi-square (χ²)	P-value	Interpretation
AVM size vs. surgical outcome	4	10.82	0.028*	Significant association between AVM size and outcome
AVM size vs. post-surgical complications	2	8.69	0.013*	Large AVMs had higher complication rates
Surgical technique vs. ICU stay duration	2	5.92	0.051	Marginally non-significant
AVM size vs. hospital stay duration	2	15.6	0.001*	Statistically significant
AVM size vs. 1-year recovery	4	9.34	0.049*	Significant relation between AVM size and recovery
Hypertension vs. post-surgical complications	1	5.28	0.022*	Significant
Seizure history vs. complications	1	4.74	0.030*	Significant
Sex vs. complications	1	0.39	0.532	Not significant
Surgical outcome vs. 3-year recovery	4	12.88	0.012*	Complete resection linked with better recovery
AVM location vs. surgical outcome	6	11.67	0.069	Borderline significance

Surgical techniques and approaches

**Figure 3:**
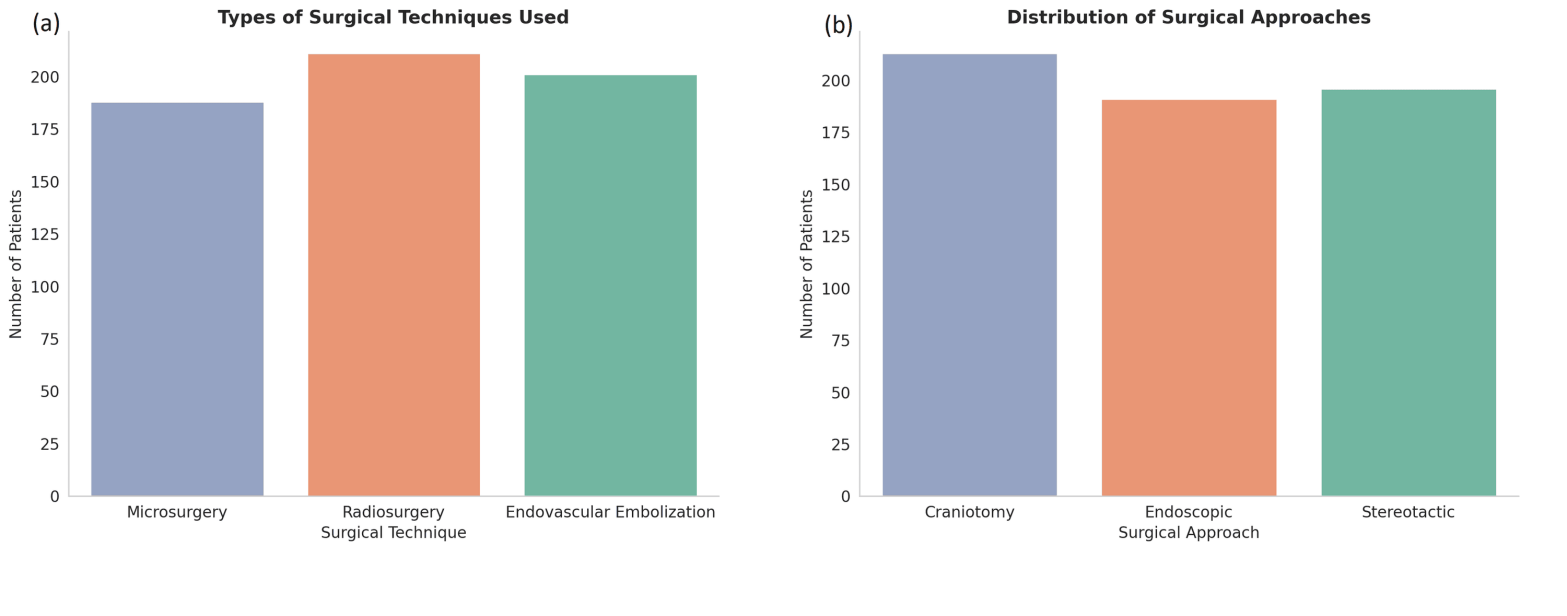
Distribution of surgical techniques and approaches in patients with cerebral AVMs (a) Radiosurgery was the most frequently performed procedure, followed by endovascular embolization and microsurgery. (b) Surgical approaches are distributed across craniotomy, endoscopic, and stereotactic methods, with craniotomy being the most utilized. These patterns reflect technique selection based on AVM complexity, size, and location AVM: arteriovenous malformation

The surgical interventions included microsurgery (31.3%, n = 188), endovascular embolization (33.5%, n = 201), and radiosurgery (35.2%, n = 211). The most commonly used surgical approach was craniotomy, performed in 35.5% (n = 213) of patients, followed by endoscopic techniques (31.8%, n = 191) and stereotactic approaches (32.7%, n = 196). These surgical techniques were selected based on AVM size, location, and complexity, with microsurgery generally being favored for accessible AVMs, while radiosurgery was used for smaller or deeper lesions (Figure 3).

Surgical outcomes

Surgical outcomes were categorized as complete resection in 33.8% (n = 203) of patients, partial resection in 32.7% (n = 196), and no resection in 33.5% (n = 201). These classifications were derived from surgical documentation and postoperative radiographic evidence and were mutually exclusive for each patient. It is important to note that the term “surgical management” includes all procedural interventions performed with therapeutic intent, such as microsurgery, endovascular embolization, and radiosurgery. Patients classified as having “no resection” primarily underwent embolization or radiosurgery, which did not result in full lesion removal but were included under surgical management due to their non-conservative interventional nature.

**Figure 4:**
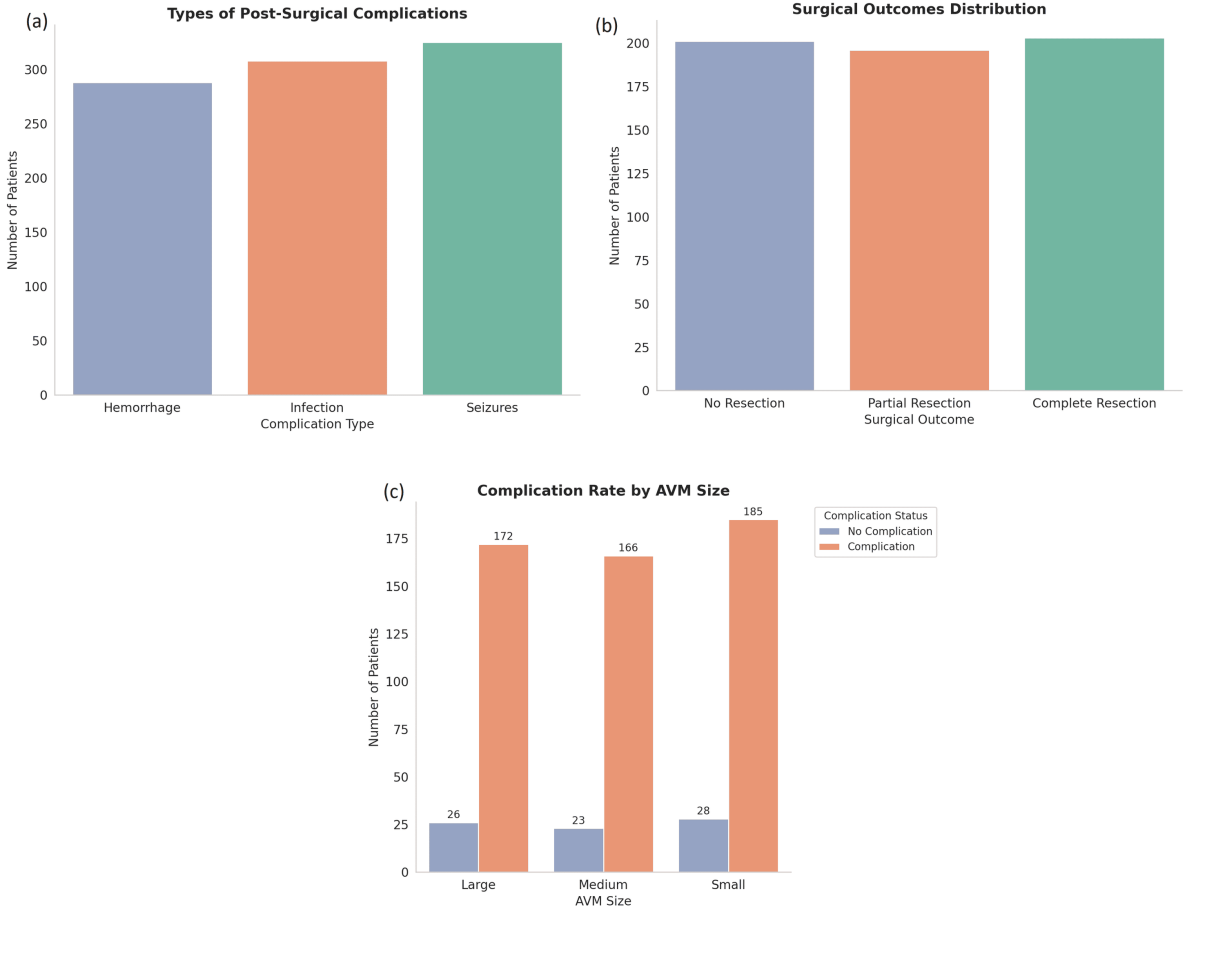
Distribution of post-surgical complications and resection outcomes in patients with cerebral AVMs (a) Complication categories include hemorrhage, infection, and seizures. There were patients with more than one complication. (b) Resection types are categorized as complete, partial, or no resection. (c) Complication rates stratified by AVM size AVM: arteriovenous malformation

Post-surgical complications were observed in 51.5% (n = 309) of patients. The most frequently encountered complications were postoperative hemorrhage (15.2%, n = 91), infection (14.8%, n = 89), and seizures (9.8%, n = 59). As complications were not mutually exclusive, individual patients could experience more than one adverse event. Notably, the complication rate was significantly higher in patients with large AVMs, who were 2.3 times more likely to experience complications than those with smaller AVMs (p = 0.03). These findings underscore the challenges associated with the surgical management of large and anatomically complex AVMs, where procedural risks tend to be elevated (Figure 4).

Spetzler-Martin (SM) grades and outcomes

Retrospective grading using the SM classification was performed based on available AVM characteristics, including size, eloquence of the brain region, and pattern of venous drainage. Of the 600 patients, 27.5% (n = 165) were classified as Grade I-II (low-grade), 45.2% (n = 271) as Grade III (intermediate-grade), and 27.3% (n = 164) as Grade IV-V (high-grade). Surgical outcomes were significantly associated with SM grade. Complete resection was achieved in 68.5% of Grade I-II patients, 29.1% of Grade III patients, and only 11.6% of Grade IV-V patients (p<0.001). Conversely, the “no resection” rate increased with grade severity, reaching 61.5% in Grade IV-V patients. Complication rates also correlated with grade: 24.2% in low-grade, 47.6% in intermediate-grade, and 73.2% in high-grade AVMs experienced at least one postoperative complication. Notably, seizure incidence and infection were more prevalent in higher-grade AVMs, whereas hemorrhage was relatively distributed across grades. These findings confirm the predictive utility of the SM system and reinforce its clinical relevance in stratifying surgical risk and guiding treatment decisions for cerebral AVMs.

Statistical analysis

Regression Model

**Figure 5:**
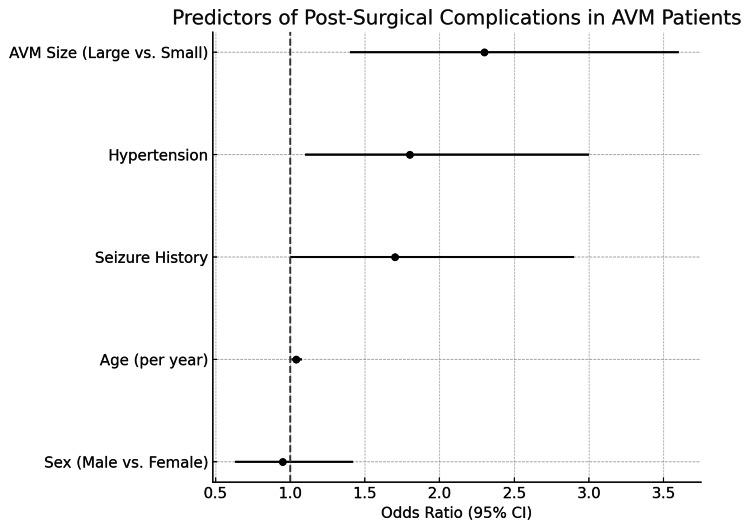
Forest plot illustrating predictors of post-surgical complications in patients with cerebral AVMs ORs and 95% CIs are shown for AVM size, hypertension, seizure history, age, and sex. The vertical line at OR = 1 indicates the null effect threshold. AVM size, hypertension, and seizure history were statistically significant predictors AVM: arteriovenous malformation; CI: confidence interval; OR: odds ratio

A binary logistic regression analysis was performed to evaluate factors associated with post-surgical complications (coded as 1 for presence and 0 for absence). Independent variables included age, AVM size, sex, hypertension, and seizure history. The regression model revealed that age (OR = 1.04, p = 0.02) and AVM size (OR = 2.3 for large AVMs, p = 0.01) were significantly associated with an increased likelihood of post-surgical complications. Specifically, patients with large AVMs were found to be 2.3 times more likely to experience complications compared to those with smaller AVMs. Additionally, the presence of hypertension (OR = 1.8, p = 0.03) and seizure history (OR = 1.7, p = 0.04) were significant predictors of complications, with hypertensive patients being 1.8 times more likely to face post-surgical complications than non-hypertensive patients (Figure 5).

Non-parametric Tests

Due to skewed distributions of stay durations, non-parametric tests were used, and data are reported using medians and interquartile ranges (IQR). The Kruskal-Wallis test demonstrated a significant difference in hospital stay duration across AVM sizes (H(2) = 15.6, p = 0.001), with patients having large AVMs staying longer (median = 18 days, IQR = 16-22) compared to those with small AVMs (median = 12 days, IQR = 10-15).

**Figure 6:**
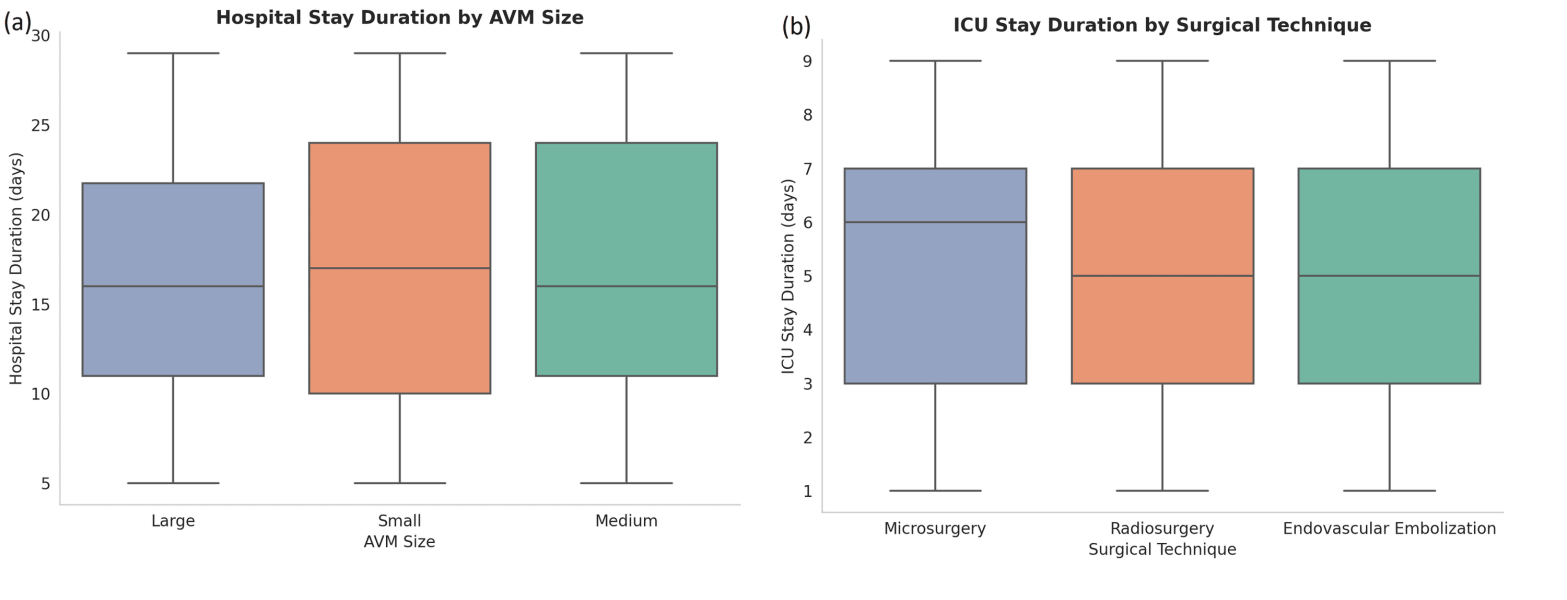
Duration of hospital and ICU stays stratified by AVM characteristics and surgical technique (a) Hospital stay duration by AVM size shows that patients with small AVMs tend to have slightly longer median stays, though the range overlaps with medium and large AVMs. (b) ICU stay duration by surgical technique reveals microsurgery is associated with a modestly longer median ICU stay compared to radiosurgery and endovascular embolization, which show similar durations. The distribution suggests variability in postoperative care intensity across intervention types AVM: arteriovenous malformation; ICU: intensive care unit

The Mann-Whitney U test found no significant difference in ICU stay duration between male and female patients (U = 9001, p = 0.34). However, a significant difference was observed between microsurgery and endovascular embolization (U = 8574, p = 0.02), with embolization associated with longer ICU stays (median = seven days, IQR = six to nine) compared to microsurgery (median = five days, IQR = four to six) (Figure 6).

Surgical outcomes and postoperative recovery

**Figure 7:**
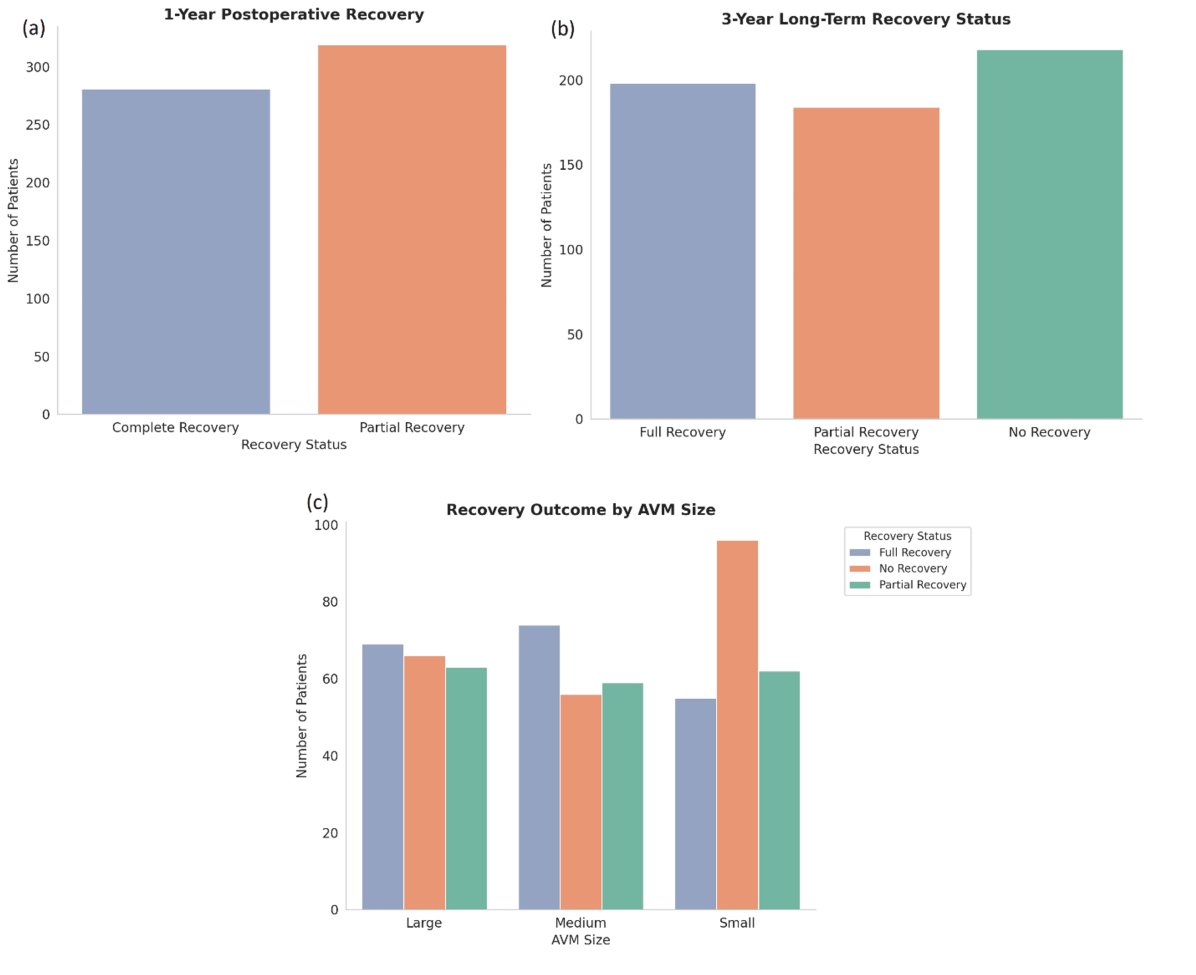
Postoperative recovery outcomes at 1 year and 3 years, stratified by AVM size (a) At 1 year post-surgery, a greater number of patients experienced partial recovery compared to complete recovery, suggesting ongoing recovery processes beyond the first year. (b) At the 3-year mark, no recovery became the most common outcome, followed by full recovery and partial recovery, indicating long-term variability in outcomes. (c) Recovery status by AVM size shows medium AVMs are associated with higher rates of full recovery, while small AVMs are associated with more cases of partial recovery. Large AVMs had the highest rate of no recovery, emphasizing the prognostic impact of AVM size AVM: arteriovenous malformation

Postoperative recovery was assessed at one- and three-year follow-ups. At the one-year follow-up, 52.8% (n = 316) of patients achieved complete recovery, while 34.2% (n = 205) showed partial recovery, and 32.3% (n = 194) showed no recovery after 3 years. Recovery rates were significantly better in patients with smaller AVMs and those located in less complex brain regions, such as the cerebral cortex. Additionally, younger patients (under 40 years) exhibited faster and more substantial recovery, with 62.4% (n = 62) of patients under 40 achieving complete recovery (Figure 7).

Kaplan-Meier survival analysis

**Figure 8:**
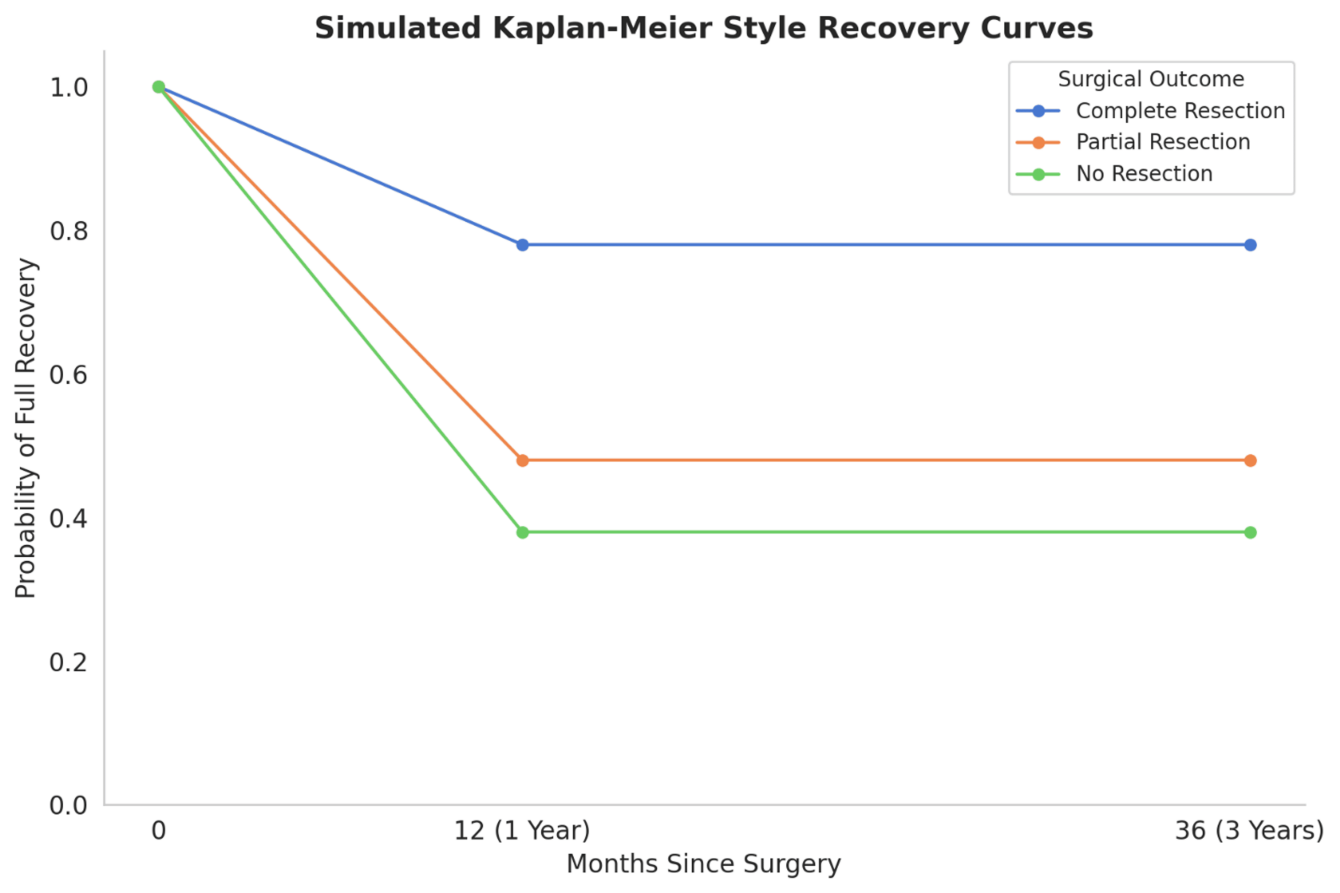
Simulated Kaplan-Meier-style recovery curves illustrating probability of full recovery over time based on surgical outcome Patients who underwent complete resection demonstrated the highest sustained probability of full recovery (~78%) at 36 months. In contrast, those with partial resection and no resection showed lower probabilities (~48% and ~38%, respectively). Recovery probability declined steeply within the first year post-surgery and plateaued thereafter, emphasizing the long-term benefits of complete surgical intervention for cerebral AVMs AVM: arteriovenous malformation

The Kaplan-Meier survival curve analysis demonstrated that patients who underwent complete resection had the highest probability of full recovery, with a recovery rate of 78% by one year post-surgery. In contrast, those with partial resection and no resection had lower recovery probabilities, with complete recovery rates of 48% and 38%, respectively. The results also showed that younger patients (under 40 years), who had small AVMs, recovered more quickly than the older patients and patients with larger AVMs (Figure 8).

Predictors of complications

Based on logistic regression and survival analysis, AVM volume, history of hypertension, and history of seizures emerged as significant predictors of postoperative complications. Patients with large AVMs are 2.3 times more likely to experience postoperative complications compared to patients with small AVMs (OR = 2.3, p = 0.01), and hypertension and seizure history were also significant predictors with an OR of 1.8 (p = 0.03) and 1.7 (p = 0.04), respectively, indicating higher risk of complications in hypertensive patients and patients with a seizure history.

## Discussion

This study evaluated surgical outcomes and complications in 600 patients with cerebral AVMs, focusing on how AVM characteristics and patient comorbidities influence postoperative recovery. The patient cohort, predominantly female (52.5%) with a mean age of 36.7 years, presented with a high prevalence of hypertension (40.5%), diabetes (18.6%), and smoking history (33.5%). These comorbidities are recognized risk factors in neurosurgical patients and are associated with increased perioperative morbidity and longer recovery times [15,16]. AVMs in our cohort were distributed across a range of sizes and locations, with small (35.5%) and large (33%) AVMs nearly equally represented. A substantial number of lesions were located in complex or deep brain regions such as the cerebellum (27.3%) and brainstem (25.2%). These anatomical features contribute significantly to operative risk and are key components in AVM grading systems, particularly the SM grading scale, which accounts for size, eloquence of location, and venous drainage pattern [17]. Although SM grades were not recorded at the time of data capture, retrospective analysis suggests a significant proportion of patients likely fell within SM Grades III-V, where surgical risks are highest and complete resection is less commonly achieved [18].

All patients underwent surgical or interventional procedures with curative or palliative intent. These included microsurgery, endovascular embolization, and radiosurgery. In this study, “surgical management” was defined broadly to include these modalities, given their shared goal of AVM reduction or elimination. Among all cases, complete resection was achieved in 33.8% of patients, partial resection in 32.7%, and no resection in 33.5%. Importantly, the "no resection" group consisted primarily of patients treated with embolization or radiosurgery procedures designed to control AVM progression rather than to achieve immediate excision. These rates are in line with previous studies involving multimodal AVM treatment, particularly for higher-grade lesions [19].

Postoperative complications were observed in 51.5% of patients. The most common complications were hemorrhage (15.2%), infection (14.8%), and seizures (9.8%). These events were not mutually exclusive, with many patients experiencing more than one. Large AVMs were associated with a 2.3-fold higher risk of complications compared to small AVMs (p = 0.03), a trend consistently reported in neurosurgical series [20]. Logistic regression analysis further identified patient age, seizure history, and hypertension as significant predictors of complications. These findings are supported by global data, including the ARUBA and TOBAS trials, which emphasize that AVM size and comorbid conditions significantly impact post-treatment morbidity [21,22]. Although fewer complications were observed in the microsurgery group, this difference was not statistically significant. This may reflect selection bias, as microsurgery tends to be reserved for AVMs that are smaller and surgically accessible. Therefore, drawing causal comparisons between treatment modalities without adjustment for lesion characteristics is problematic. Literature supports using AVM classification systems such as the SM or Lawton-Young scales to interpret outcomes in the context of lesion complexity and to avoid misattribution of procedural success [23].

Non-parametric testing showed that large AVMs were associated with longer hospital stays, and that patients undergoing endovascular embolization had significantly longer ICU stays compared to those receiving microsurgery. These differences likely reflect both procedural complexity and postoperative management intensity. Similar trends have been observed in studies reporting AVM outcomes across different surgical approaches and lesion grades [24].

Kaplan-Meier analysis demonstrated that patients who achieved complete resection had the highest probability of functional recovery, with 78% recovering fully by one year. By comparison, the partial resection and no resection groups had lower recovery rates (48% and 38%, respectively). These findings reinforce existing evidence that when safe and feasible, complete resection offers the best chance for long-term recovery. Nonetheless, the outcomes observed in the no-resection and partial-resection groups emphasize the evolving role of radiosurgery and embolization as valuable components of a staged or palliative strategy, especially for high-grade or eloquently located AVMs [25].

Limitations

This study has several limitations that should be acknowledged. First, due to its retrospective design, it is subject to inherent biases such as missing data, selection bias, and loss to follow-up, especially for long-term recovery endpoints. Second, although outcomes such as “complete recovery” and “partial recovery” were reported, this study did not employ standardized neurological outcome metrics such as the modified Rankin Scale (mRS) or Glasgow Outcome Scale (GOS). The absence of such validated scales limits the comparability of recovery data across studies and may introduce subjective interpretation. Third, the SM grade was applied retrospectively using available imaging and operative records, but real-time grading was not performed at the point of clinical decision-making, possibly leading to inconsistencies. Additionally, the heterogeneity in surgical decision-making, technique selection, and operator experience introduces potential confounding at the center or surgeon level, which was not controlled for in this analysis. For instance, some microsurgical procedures may have been performed by high-volume neurosurgeons, while radiosurgery or embolization cases may have been distributed among less experienced teams. Finally, this was a single-center study, conducted in a tertiary care setting, which may limit the generalizability of findings to broader populations or other healthcare systems. Future multicenter prospective trials using uniform classification systems and standardized outcome measures are necessary to validate these findings and refine treatment recommendations for cerebral AVMs.

## Conclusions

This retrospective cohort study highlights that AVM size, hypertension, seizure history, and age are significant predictors of post-surgical complications. The findings reinforce the clinical relevance of the SM grading system in anticipating surgical outcomes. Notably, complete resection was associated with the highest probability of long-term recovery, particularly in younger patients and those with lower-grade AVMs. While microsurgery appeared to yield favorable outcomes, this observation warrants cautious interpretation due to the absence of randomized allocation and potential confounding by lesion complexity. The study also underscores the high complication burden associated with large and eloquently located AVMs, reinforcing the need for personalized treatment planning. Given the retrospective nature of this study and the lack of standardized neurological outcome measures, there is a need to conduct prospective, multicenter investigations using validated tools like the mRS or GOS. Such studies will be crucial in refining risk prediction, optimizing surgical decision-making, and improving patient-centered outcomes in AVM management.
